# Deep Eutectic Solvents: Properties and Applications in CO_2_ Separation

**DOI:** 10.3390/molecules28145293

**Published:** 2023-07-08

**Authors:** Iwona Cichowska-Kopczyńska, Bartosz Nowosielski, Dorota Warmińska

**Affiliations:** 1 Department of Process Engineering and Chemical Technology, Faculty of Chemistry, Gdańsk University of Technology, 80-233 Gdańsk, Poland; 2 Department of Physical Chemistry, Faculty of Chemistry, Gdańsk University of Technology, 80-233 Gdańsk, Poland; bartosz.nowosielski@pg.edu.pl (B.N.); dorota.warminska@pg.edu.pl (D.W.)

**Keywords:** carbon dioxide, deep eutectic solvent, absorption, membrane, separation, solubility

## Abstract

Nowadays, many researchers are focused on finding a solution to the problem of global warming. Carbon dioxide is considered to be responsible for the “greenhouse” effect. The largest global emission of industrial CO_2_ comes from fossil fuel combustion, which makes power plants the perfect point source targets for immediate CO_2_ emission reductions. A state-of-the-art method for capturing carbon dioxide is chemical absorption using an aqueous solution of alkanolamines, most frequently a 30% wt. solution of monoethanolamine (MEA). Unfortunately, the usage of alkanolamines has a number of drawbacks, such as the corrosive nature of the reaction environment, the loss of the solvent due to its volatility, and a high energy demand at the regeneration step. These problems have driven the search for alternatives to that method, and deep eutectic solvents (DESs) might be a very good substitute. Many types of DESs have thus far been investigated for efficient CO_2_ capture, and various hydrogen bond donors and acceptors have been used. Deep eutectic solvents that are capable of absorbing carbon dioxide physically and chemically have been reported. Strategies for further CO_2_ absorption improvement, such as the addition of water, other co-solvents, or metal salts, have been proposed. Within this review, the physical properties of DESs are presented, and their effects on CO_2_ absorption capacity are discussed in conjunction with the types of HBAs and HBDs and their molar ratios. The practical issues of using DESs for CO_2_ separation are also described.

## 1. Introduction

One of the main objectives of current global research is to develop materials and methods aimed at reducing emissions of acid and toxic gases, especially carbon dioxide. One of the most common technologies used to remove CO_2_ is its absorption with aqueous amine solutions, mainly a 30% aqueous solution of monoethanolamine (MEA). However, other amine absorbents can be considered, particularly those including hydroxyamines such as aminomethylpropanol (AMP) or 2-piperidineethanol (2-PPE) which, compared to aqueous MEA solutions, require less heat at the regeneration stage, resulting in a reduction in the process cost. However, new alternative solvents will be indispensable to make the process more efficient, still less expensive, and more environmentally friendly.

Over the last few years, a lot of attention has been paid to ionic liquids (ILs). These solvents are characterized by a low melting point, low vapor pressure, and relatively high solubility of CO_2_. However, research has shown that a vast majority of ILs absorb carbon dioxide physically, which ensures relatively low costs for solvent regeneration, but at the same time limits capacity and requires high operating pressures for the removal of carbon dioxide. Serious disadvantages of ionic liquids used on a technical scale are their high price, relatively high viscosity, toxicity, and corrosivity.

**Figure 1:**
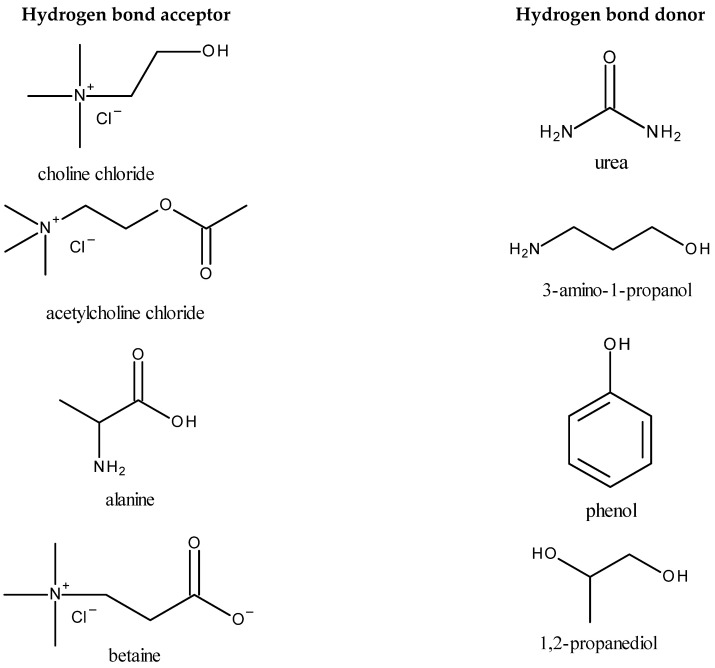

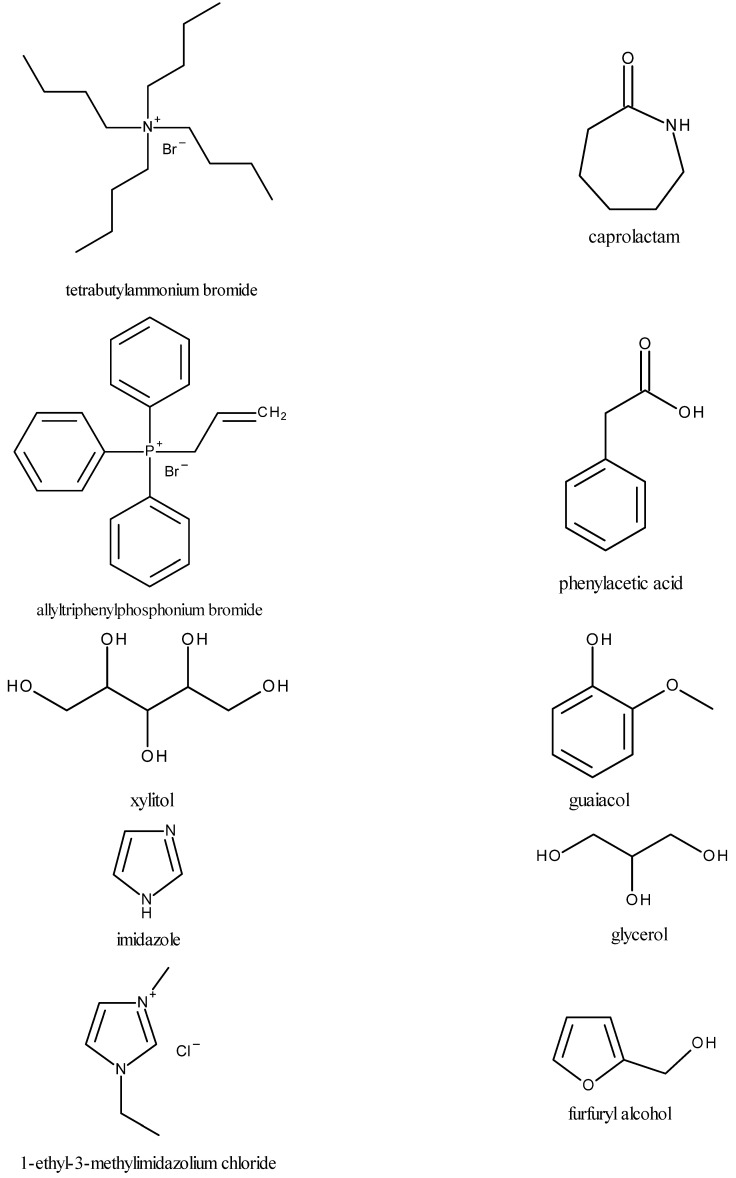

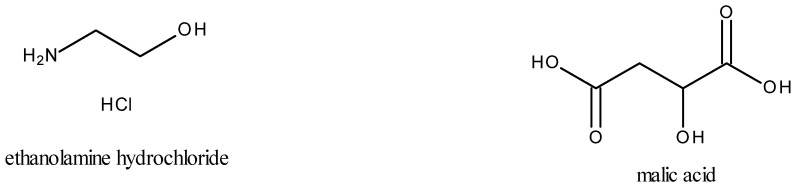
Examples of commonly used HBAs and HBDs.

An alternative to ILs is using deep eutectic solvents (DESs), which were originally introduced as systems formed from a mixture of two or more Lewis acids and bases or Brønsted–Lowry acids and bases, and which have a lower freezing point compared to the starting constituents. In recent years, the definition of a DES was made more precise and it has been established that the term “deep eutectic solvent” can only be assigned to a eutectic mixture for which the eutectic point temperature is lower than that calculated from the Schröder–Van Laar equation valid for an ideal liquid eutectic mixture [1]. However, in practice, most researchers use the term “deep eutectic solvent” simply for the eutectic mixture. DESs are usually obtained by mixing several reagents to form a homogenous liquid. There is always a small amount of water in these solvents, which is involved in the formation of a network of hydrogen bonds. Deep eutectic solvents have similar physical properties to ionic liquids, and they are practically non-volatile and non-flammable, but additionally they are definitely cheaper and usually easier to synthesize, and they are less toxic and often biodegradable. However, compared to ILs, DESs have lower thermal stability and a lower electrochemical window [2]. The vapor pressure of eutectic liquids is higher than that of ionic liquids, but still relatively low. For an *N*,*N*-diethylethanolammonium chloride 1: 2 mixture with glycerol at temperatures from 343 to 393 K, the vapor pressure is from 2 to 60 Pa, and for mixtures with urea it is from 0.14 to 6.8 Pa [3]. Similar to ionic liquids, eutectic liquids’ physical properties can be controlled by changing the composition and ratio of their components in order to obtain properties favorable for a specific application. These properties are affected by the chemical structure of the DESs and the resulting interactions between their components, mainly the hydrogen bonds that are responsible for the physical state of deep eutectic solvents. Examples of commonly used ingredients of DESs, i.e., hydrogen bond acceptors (HBAs) and hydrogen bond donors (HBDs), are presented in Figure 1.

Taking into account the available research reports, it can be concluded that there are no highly accurate models able to predict the properties of DESs. Most of the currently available knowledge is focused on choline chloride-based DESs. Although this is the most widely used hydrogen bond acceptor, there is a whole range of DESs based on other compounds that have not been described in detail. DESs have found their place in several separation applications [4,5,6]. Hence, they are considered to be green and have been examined for the absorption and separation of carbon dioxide and other gaseous pollutants. Depending on their structure, deep eutectic mixtures can absorb carbon dioxide through physical or chemical absorption.

This present article reviews the properties that make these solvents appropriate for CO_2_ separation applications, as well as their CO_2_ absorption capacity.

## 2. Properties of Deep Eutectic Solvents

Growing interest in DESs as a new generation of solvents for various practical applications has resulted in the need for accurate and reliable knowledge about their main physical, chemical, and thermodynamic properties. Therefore, numerous scientific articles devoted to the development and characterization of DES properties have recently been added to the literature. This section describes and discusses the main physicochemical and thermodynamic properties of DESs, including those that affect their suitability for gas separation.

There are seven types of DESs that can be distinguished. The general formulas and examples of the specific types are presented in Table 1. Since most of the research carried out so far has been related to the properties and applications of mixtures of quaternary ammonium salts and HBD, this review is focused mainly on DESs of type III.

### 2.1. Freezing Point

**Figure 2:**
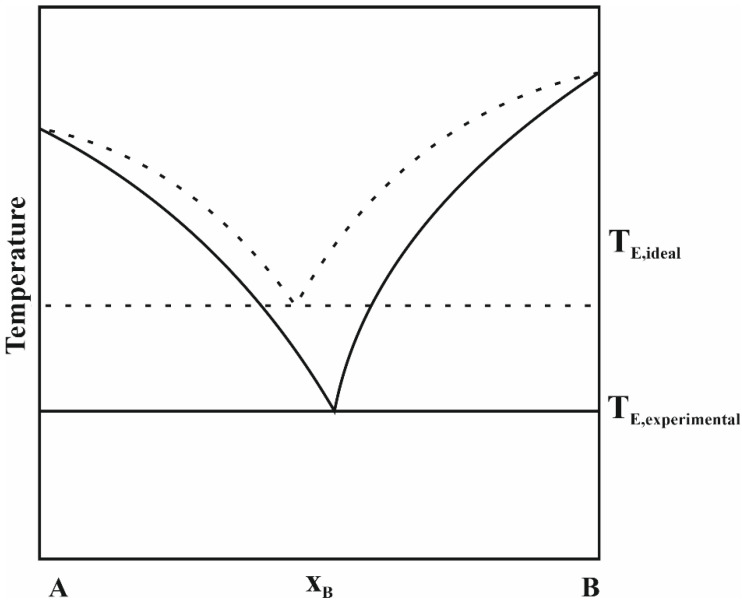
Phase diagram of deep eutectic solvent.

As mentioned above, a DES is formed by mixing two or three components capable of forming a new liquid phase with a lower freezing point than that for the ideal eutectic mixture (Figure 2) [1]. A significant depression of the freezing point is observed mainly due to the hydrogen bond interactions between the hydrogen bond acceptor and the hydrogen bond donor. Although the number of DESs reported in the literature is huge, the number of DESs with the freezing point lower than room temperature is still limited. In general, the freezing point of a DES is influenced by the nature of its constituents and their molar ratio. However, no clear correlation between the melting points of individual components and the freezing points of the corresponding DESs has been found. Among all the choline chloride-based DESs studied, lower melting points were obtained for DESs consisting of polyols such as glycerol or ethylene glycol [7,8]. However, Shahbaz et al. recorded a very low melting point for 2,2,2-trifluoroacetamide, which confirms a very large contribution of the HBD to lowering the melting point by forming a hydrogen bond [8]. It has been established that the nature of the HBA anion also affects the DES freezing point. For example, Abbott et al. studied DESs based on urea and they concluded that DESs consisting of urea and a choline salt have freezing points decreasing in the order $F^{-}>{\mathrm{NO}}_{3}^{-}>{\mathrm{Cl}}^{-}>{\mathrm{BF}}_{4}^{-}$ [9]. Moreover, in their article, the HBA effect on the freezing point of the DESs was investigated. The researchers found that mixing urea with different salts in the same ratio (1:2) results in liquids that exhibit very different freezing points from −38 to 113 °C.

The influence of the molar ratio of HBAs and HBDs on the freezing point of a DES has been studied by many researchers and it has been demonstrated that, depending on the mixture, it can be significant. For example, when methyl triphenyl phosphonium bromide was mixed with glycerol in a molar ratio of 1:3 or 1:4, the resulting DESs exhibited freezing points of −5.5 and 15.6 °C, respectively [10].

Abbott et al. suggested that the depression of the melting point is due to the lattice energy of the DES, and the entropy changes are caused by the formation of a liquid phase and the interaction between HBAs and HBDs [11]. Based on the analysis of 13 choline chloride DESs, researchers have tried to show a correlation between the freezing point depression and the experimentally measured enthalpy of hydrogen bond formation. However, the discovered correlation was not satisfactory [12]. Marcus tried to compare the standard entropies of DES formation with the freezing point depression but did not identify any general correlation [13].

Pontes et al. used the perturbed-chain statistical associating fluid theory (PC-SAFT) for modelling the phase behavior of DES systems [14]. They observed a compatibility with the experimental data, but this method requires a number of molecules and mixing parameters fitted from experimental data. Thus, this model has a limited predictive capability for novel systems. Garcia et al. proposed a quantitative structure–activity relationship (QSAR) predictive model for choline chloride-based DESs and obtained high predictive ability as shown by the cross-validated R^2^ values of 0.93 [15]. Recently, a conductor-like screening model for realistic solvation (COSMO- RS) applying experimentally independent molecular descriptors has been proposed by Song et al. [16]. Using 35 choline chloride-based DESs, they found a reliable multilinear relationship between the freezing point depression of DESs and the molecular volume descriptor and their molecular descriptors associated with hydrogen bond interactions.

### 2.2. Vapor Pressure

Despite a very limited amount of data on the vapor pressure of DESs, there is general agreement that the vapor pressures of pure deep eutectic solvents at ambient temperatures are low or even negligible, but higher than those of ionic liquids. Recently, Ravula et al. studied the vapor pressure of fluids presenting low-volatility and found that they vary in the following order: short-chain PEGs > long-chain PEGs > DESs > protic ILs > polymeric ILs > aprotic ILs > dicationic ILs [17].

Experimental measurements of the vapor pressures of deep eutectic solvents have so far been taken only at 40–120 °C, considerably above ambient temperatures. Boisset et al. determined the vapor pressure at 40 °C for a DES consisting of *N*-methylacetamide and lithium bis(trifluoromethylsulfonyl)-imide to be 20 Pa [18]. Shahbaz et al. found that at 100 °C the vapor pressures of reline, glyceline, diethylethanolammonium chloride:urea, *N,N*-diethylethanolammonium chloride:glycerol, and methyltriphenylphosphonium bromide:glycerol are equal to 1.33, 11.6, 1.8, 16.9, and 9.9 Pa, respectively [2]. Recently, Dietz et al. measured the vapor pressures of hydrophobic DESs at 100 °C and obtained values in a range from 55.5 Pa for decanoic acid:lidocaine to 540.9 Pa for decanoic acid:menthol [19].

### 2.3. Density

Most DESs have a density between 1.0 and 1.35 g·cm^−3^ at 25 °C [20]. However, DESs containing metal salts, such as ZnCl_2_, have slightly higher densities, in the range of 1.3 to 1.6 g·cm^−3^, whereas hydrophobic DESs exhibit a density lower than that of water [21,22]. It is certain, however, that DES densities are higher than those of their individual starting components. According to the principle of the hole theory, mixing DES components reduces the average hole’s radius and thus increases DES density relative to that of the individual constituents.

In general, the influence of the amount of HBDs on the density of a DES depends on the molecular characteristics of the HBD used. For most DESs, their density decreases as the amount of HBDs increases [23,24,25,26,27]. However, in the case of DESs, where there is a strong association between HBD molecules, an increase in DES density with the increasing amount of HBDs was observed. Abbott et al. reported the densities of DESs with different molar ratios of choline chloride to glycerol and concluded that as the ratio of HBDs decreased, DES density increased [28].

The increasing number of hydroxyl groups in HBDs, resulting in the formation of additional H-bonds and possibly reducing the available free volume, leads to the higher density of DESs. The results obtained by Basaiahgari et al. showed that ethylene glycol-based DESs have lower densities compared to glycerol-based DESs for both benzyltrimethylammonium and benzyltributylammonium chloride as HBA [29]. The same effect was observed by Mjalli et al. for similar choline chloride-based DESs [30].

According to the literature, the increase in DES density is also caused by the presence of additional carboxylic groups as well as additional ether bonds in the HBDs. For example, in the case of DESs based on choline chloride, the density of a DES consisting of oxalic acid was higher than that of a DES consisting of glycolic acid [31]. Basaiahgari et al. reported the densities of DESs consisting of ethylene, diethylene, and triethylene glycol as HBDs and benzyltrimethylammonium chloride as the HBA to be equal to 1.101, 1.110, and 1.117 g·cm^−3^ at 25 °C, respectively [29].

It has been established that the density of DESs decreases with the increasing chain length of the HBDs or HBAs. The results obtained by Florindo et al. show that DESs composed of glutaric acid or levulinic acid have lower density values compared to those observed for DESs based on oxalic or glycolic acids [31]. Wang et al. reported that the density of ethylene glycol-based DESs consisting of tetraethylammonium bromide, tetrapropylammonium bromide, and tetrabutylammonium bromide were 1.1596, 1.1121, and 1.0762, respectively [32].

Another factor affecting DES density is the type of salt they consist of. Shahbaz et al. observed that ammonium-based DESs have lower densities than phosphonium-based DESs [24]. Moreover, bromide salts form denser DESs than chloride ones [23].

In general, an increase in temperature causes a linear decrease in density [33]. However, Yadav et al. found that the decrease in density with increasing temperature of a DES composed of choline chloride and glycerol in a 1:2 mole ratio follows a quadratic expression [34]. Temperature-dependent density measurements for DESs can be used to estimate their isobaric thermal expansion coefficients, which can quantify DESs’ free volume [29].

Several attempts at predicting deep eutectic solvents’ densities have been reported in the literature so far. Shahbaz et al. applied artificial intelligence and group contribution methods in order to predict the densities of DESs consisting of ethylene glycol and glycerol as HBDs and choline chloride, diethylethanolammonium chloride, and methyltriphenylphosphonium bromide as HBAs [24]. Mjalii introduced mass connectivity index (MCI)-based density prediction for a set of 20 deep eutectic solvent systems comprising various salts and hydrogen bond donors [35]. Nowosielski et al. used the Rackett equation modified by Spencer and Danner and the MCI-based density model to predict the density of DESs based on 3-amino-1-propanol [23]. Finally, recently, Haghbakhsh et al. presented group contribution and atomic contribution models for predicting the density of various types of DESs by simply decomposing the molecular structure into a number of predefined groups or atoms [36].

### 2.4. Viscosity

The viscosity of deep eutectic solvents varies significantly at room temperature. The lowest viscosity values were observed for DESs based on ethylene glycol, ethanolamine, or acetic acid [37,38]. According to Mjalii and Naser, a mixture of choline chloride and ethylene glycol in a molar ratio of 1:2 has a viscosity of 40 mPa·s at 25 °C [39]. The highest viscosities are those of DESs containing derived sugars or amino acids [40,41]. A DES consisting of sorbitol and choline chloride in a molar ratio of 1:12 has a viscosity of 19,470 mPa·s at 25 °C [42]. However, most DESs show relatively high viscosities at room temperature (>100 mPa·s) [20].

Since viscosity is related to the free volume and the probability of finding holes of suitable dimensions for solvent molecules or ions to enter, this property depends on the size of the DES constituents. The high viscosity of DESs also results from the presence of hydrogen bonds as well as electrostatic and van der Waals interactions between the individual components of the DESs. Thus, the viscosity depends on the chemical nature of the components, their molar ratio, water content, and temperature.

In general, increasing the amount of HBDs reduces the viscosity of a DES [25]. However, in the case of DESs based on hydrogen bond donors with a strong cohesive energy, due to the presence of the intermolecular hydrogen bond network, the opposite effect is observed. At 25 °C, the viscosities of choline chloride and glycerol mixtures with molar ratios of 1:4, 1:3, and 1:2 were 350, 320, and 259 mPa·s, respectively [28].

The viscosity of DESs decreases considerably with increasing temperature. For example, for choline chloride:urea (1:2) and choline chloride:glucose (2:1), the viscosity decreases from 750 to 95 mPa·s and from 7992 to 262 mPa·s, respectively, at 25 and 55 °C [43]. The dependence of DESs’ viscosity on temperature is described in the literature by Arrhenius or Vogel–Fulcher–Tamman (VFT) equations [20]. Mjalli and Naser proposed a model for the viscosity of choline chloride-based DESs taking into account not only the temperature, but also the composition of the mixture. According to their results, the model based on the Arrhenius equation is more accurate for less viscous liquids, while the VFT-based viscosity model fits experimental viscosities also for more viscous DESs in a wide temperature range [39].

It has been established that DESs’ viscosity drastically decreases with the water content. This property seems to be the main reason for the differences in the literature values of viscosity for any given deep eutectic solvent, in some cases even within a factor of two. According to Florindo et al., a highly viscous DES such as choline chloride:oxalic acid (1:1) is capable of capturing water from the atmosphere up to 19.40 wt%, which reduces its viscosity from 5363 to 44.49 mPa·s [31].

In 2018, Haghbakhsh et al. modelled the viscosity of deep eutectic solvents following the free volume theory coupled with equations of state [44]. The results showed that the free volume theory with both the CPA and PC-SAFT EoSs (total AARD% of 2.7% and 2.7%, respectively) gives reliable and highly accurate results with respect to the corresponding experimental viscosity values of 27 DESs. Both models also showed good compliance with the temperature trends of viscosity for the investigated DESs. Furthermore, the effect of changing HBD ratios for a fixed HBA was correctly estimated.

More recently, Benguerba et al. proposed a new mathematical model for predicting amine-based DESs’ viscosities using the quantitative structure–property relationship (QSPR) approach. To develop this model, a combination of the multilinear regression (MLR) and the artificial neural networks (ANN) methods was applied. The results show that the proposed models are able to predict DESs’ viscosities with very high accuracy, i.e., with an R^2^ value of 0.9975 in training and 0.9863 for validation using the ANN model, and an R^2^ value of 0.9305 for the MLR model [45].

### 2.5. Surface Tension

The surface tension values of DESs are higher than those of most molecular solvents and exhibit a wide range of changes from 23.9 mN·m^−1^ for tetrabutylammonium bromide:1-nonanol (1:4) to 75.2 mN·m^−1^ for choline chloride: D-glucose (2.5:1) at 20 °C [22,46].

Significant roles in the surface tension values of DESs are played by the nature and length of the alkyl chain of HBAs, the molar ratio of HBA:HBD, and temperature. A cation containing a hydroxyl group leads to the formation of a DES with high surface tension. For instance, according to Omar and Sagedhi, the surface tension of choline chloride:pyrogallol (1:1) is equal to 68.0 mN·m^−1^, while for DESs based on the same HBD but with tetrabutylammonium bromide as the HDA, the surface tension is 41.0 mN·m^−1^ at 20 °C [47]. The results obtained by the same authors further demonstrated that in the case of tetraalkylammonium-based DESs, an increase in the chain length leads to an increase in the surface tension in the following order: tetraethylammonium bromide:pyrogallol < tetrapropylammonium bromide:pyrogallol < tetrabutylammonium bromide:pyrogallol.

According to the literature, surface tension increases with a decrease in the molar fraction of the salt due to the strengthening of HBD hydrogen bonding. Thus, the surface tension of DESs consisting of choline chloride and lactic acid decreases with increasing salt concentration, due to the disturbance of the hydrogen bond network of lactic acid [48]. Hayyan et al. analyzed the effect of increasing the molar amount of choline chloride in DESs containing D-glucose. At 20 °C, with a 1:1 molar ratio of choline chloride to D-glucose, the surface tension (70.4 mN·m^−1^) was lower compared to that for a 2.5:1 molar ratio (75.0 mN·m^−1^) [46]. For all the studied DESs, the surface tensions showed an inversely proportional linear correlation with the temperature [39,46,47,48,49].

There are reports of successful prediction of the surface tension of DESs using the group contribution and atomic contribution models and the Guggenheim empirical equation [30,34].

### 2.6. Electrical Conductivity

Due to their relatively high viscosity, most DESs exhibit ionic conductivities lower than 1 mS·cm^−1^ at room temperature. The exception is a DES composed of ethylene glycol and choline chloride, the conductivity of which is equal to 7.61 mS·cm^−1^ at 20 °C [37].

The conductivity of DESs generally increases significantly as the temperature increases, and the Arrhenius-like equation or the Vogel–Fulcher–Tammann (VFT) expression can be used to predict the conductivity behavior of DESs [20].

The conductivities of DESs are dependent on both the HBD and the nature of the salt. Basaiahgari et al. showed that DESs based on benzyltrimethylammonium chloride and benzyltributylammonium chloride have conductivities relatively lower than those of their choline chloride analogues, which can be attributed to their high viscosity values [29].

Generally, an increase in the amount of salt in a DES causes an increase in its conductivity. Such a phenomenon was observed by Abbott et al. for a DES consisting of choline chloride and glycerol [28]. However, this behavior is not true for all the DESs for which the conductivity–salt concentration trend evolves through a maximum (e.g., choline chloride + ethylene glycol) or is decreasing (e.g., tetrabutylammonium chloride + ethylene glycol) [15].

The relationship between viscosity and conductivity is commonly analyzed in the literature using Walden plots, in which the molar conductivity (Λ, calculated from conductivity and density) and the fluidity (the inverse of viscosity) are plotted on log−log scales and compared with an ideal line obtained for a 0.01 M KCl aqueous solution. According to the literature data, Walden plots indicate that almost all DESs lie below the “ideal” Walden line [15]. However, Mjalii et al. observed a positive deviation for aliphatic-based DESs such as tetrabutylammonium chloride:L-isoleucine, tetrabutylammonium chloride:proline, and tetrabutylammonium chloride:L-valine, which indicates a high ionic pairing between tetrabutylammonium chloride and amino acids in these DESs [49].

Overall, the literature results show that DESs display larger deviations from the ideal reference line than most ILs, which can be explained by considering the fact that the migrating species are ions for ILs and ions + HBD complexes for DESs. Therefore, it can be concluded that the conductivity of DESs is determined not only by their viscosity, but also by the size of the ions.

The development of predictive methods for DES conductivity is very limited in the literature. Bagh et al. used the artificial neural network (ANN) approach to study the electrical conductivity of ammonium- and phosphonium-based DESs and they obtained an absolute relative deviation of 4.4%, which may be considered satisfactory [50]. The hole theory was also applied by Abbott et al. to predict DESs’ conductivity, and although good results were obtained in some cases (e.g., choline chloride:ethylene glycol), poor predictions were reported for others (e.g., choline chloride:glycerol) [37].

### 2.7. Solvatochromic Parameters

Solvent polarity is commonly assessed using the polarity scale of Dimroth and Reichardt, *E_T_*(30), which is important for understanding the solubilizing power. Other parameters are the Kamlet–Taft π*, which measures the combined polarity and polarizability of solvents, α, which measures their hydrogen bond donation ability, and β, which measures their hydrogen bond acceptance ability.

The values that have so far been reported in the literature for these solvatochromic parameters for deep eutectic solvents are generally between those of methanol and water among the common solvents and commensurate with those of ionic liquids [20]. This means that deep eutectic solvents are highly polar and polarizable, and that they have good hydrogen bond donation and acceptance abilities toward solutes.

In general, the number of publications related to DES solvatochromic parameters is limited. Abbott et al. determined the polarities of choline chloride:glycerol DESs of different molar ratios based on several parameters, revealing a linear polarity increase with increasing choline chloride concentration [28]. Pandey et al. employed solvatochromic probes to examine the polarities of four DESs based on combinations of choline chloride with glycerol, urea, malic acid, and ethylene glycol in 1:2 molar ratios [51]. They concluded that the high polarity of these DESs was significantly influenced by the HBD nature. Among the above four combinations, choline chloride:glycerol exhibited the highest *E_T_*(30) value. Moreover, Pandey et al. investigated the effect of temperature and the addition of water on the DESs’ solvatochromic parameters. These researchers demonstrated that an increase in temperature results in a reduced H-bond donor acidity for the DESs, while no temperature effect was observed for dipolarity/polarizability and H-bond accepting basicity. It was also shown that the addition of water to the DESs caused an increase in their dipolarity/polarizability and a decrease in their H-bond accepting basicity. Teles et al. determined the solvatochromic properties of DESs formed by ammonium-based salts and carboxylic acids and concluded that the studied DESs presented a greater ability to donate and accept protons compared to most ionic liquids or organic molecular solvents [52]. Moreover, according to these authors, the high acidity of the studied DESs was mainly due to the organic acid present in the mixtures, and an increase in the alkyl side chain of both the HBA and the HBD species leads to a lower ability of the solvent to donate protons. Florindo et al. determined solvatochromic properties for two different types of DESs: those based on salts, such as choline chloride and tetrabutylammonium chloride, and those based on the neutral compound DL-menthol [53]. They found high values of hydrogen-bonding acidity for all the DESs, probably due to the organic acids present in all the systems. On the other hand, hydrogen-bonding basicity of these DESs did not vary much within the same HBA, but differed slightly in the case of DESs based on choline chloride, tetrabutylammonium chloride, or DL-menthol.

### 2.8. Other Relevant Properties

*Thermal stability*. Scarce information is available in the literature on the thermal stability of DESs. Generally, the thermal stability of these solvents is determined by the nature of the hydrogen bond donor and increases with the alkyl chain length on HBDs [54]. Zhao et al. analyzed the thermal stability of DESs consisting of a choline salt (chloride or acetate form) and glycerol [55]. They showed that the studied DESs exhibit relatively high thermal resistance while being stable up to nearly 200 °C. Florindo et al. measured the decomposition temperature of choline chloride-based DESs with several carboxylic acids (levulinic, glutaric, malonic, oxalic, and glycolic) [31]. The lowest value was observed for the DES containing malonic acid (124.68 °C) and the highest was for the DES containing glutaric acid (239.05 °C). Ghaedi et al. studied DESs based on allyltriphenyl phosphoniumbromide and where the HBDs were glycerol, ethylene glycol, diethylene glycol, and triethylene glycol. They found that, among the HBDs, glycerol had the highest thermal stability, while ethylene glycol had the lowest. The DESs followed a similar trend to that of the thermal stability of the HBDs: GL > TEG > DEG > EG [54]. The studies of Delgado et al. concluded that the thermal stability of choline chloride-based DESs, which were formed using levulinic acid, malonic acid, glycerol, ethylene glycol, phenylacetic acid, phenylpropionic acid, urea, and glucose as hydrogen bond donors, increase in the following order: ChCl:EG < ChCl:MalA < ChCl:LevA < ChCl:PhenylacA < ChCl:PhenylpropA < ChCl:GL < ChCl:Urea < ChCl:Gluc [56].

*Heat capacity.* There are only a few studies available which present experimentally measured heat capacities for some DESs. The majority of the data was provided by Naser et al., who studied deep eutectic solvents of three salts (choline chloride, tetrabutylammonium chloride, and methyltriphenylphosphonium bromide) and several HBDs [57]. Siongco et al. determined the molar heat capacities of DESs based on *N*,*N*-diethylethanolammonium chloride and ethylene glycol or glycerol, while Zhang et al. measured the *C_p_* of DESs consisting of ethylene glycol and betaine or L-carnitine [58,59]. They found that the molar heat capacity values of these DESs increase with temperature and they used different degrees of polynomials for the expression of the temperature dependence. Moreover, all the authors observed the linear relationship between the molar heat capacity and the molar mass of the DESs, which is similar to that of ILs.

In general, three models, based only on knowledge of the molecular structure of the DESs, are available to predict their heat capacities. Taherzadeh et al. developed a correlation to estimate the heat capacity of DESs as a function of temperature, molecular weight, critical pressure, and acentric factor with the resulting AARD% equal to 5.5% [60]. Haghbakhsh et al. presented group contribution (GC) and atomic contribution (AC) models which can be applied to the molar heat capacities of DESs. The AARD% for the GCM and the ACM was 3.26% and 9.93%, respectively [36]. Bunquin et al. proposed a generalized model using an artificial neural network (ANN) for predicting the heat capacity of ammonium- and phosphonium-based two-component DESs [61]. The overall average absolute relative deviation of the proposed model from the data was 0.57%.

*Refractive index.* Despite the possibility of using the refractive index as an additional tool to demonstrate hydrogen bonding in DESs, this property has not often been studied [62]. However, the research carried out thus far has shown that the *n_D_* values of deep eutectic solvents are higher than those of ethanol or acetone, but similar to common ILs [63]. Moreover, for all the studied DESs, a linear decrease in the refractive index with increasing temperature was observed. Murshid et al. investigated DESs based on diethanolamine as HBDs and found that the refractive index of all the systems studied decreases with the increase in the molar ratio of HBAs to HBDs from (1:4 to 1:6) [64]. This observation was in line with the results reported by other researchers, for example by Sanchez et al. for a DES composed of betaine and lactic acid, and by Mjalli et al. for a DES composed of tetrabutylammonium bromide and monethanolamine [25,65]. Basaiahgari et al. investigated several DESs based on benzyltrimethylammonium chloride and benzyltributylammonium chloride as HBAs, with three ethylene glycols and glycerol as HBDs, and found that the refractive index decreases in the order GLY > EG > DEG > TEG, irrespective of the HBAs [29]. A report by Su et al. investigated several DESs based on tetrabutylammonium chloride (TBAC) and varying HBDs, such as propionic acid (PA), ethylene glycol (EG), polyethylene glycol (PEG), and phenyl acetic acid (PAA) [62]. Among the tested HBDs, a DES composed of TBAC and PAA had the highest refractive index. Mjalli et al. also investigated DESs based on choline chloride as the HBA, but as HBDs they used glutamic acid, aspartic acid, and arginine, and they found that for acidic amino acids-based DESs, the refractive index values were lower than that for the corresponding basic one (arginine) [40]. Troter et al. investigated DESs based on choline chloride as HBA and obtained the following order of the refractive index: ChCl:thiourea > ChCl:urea > ChCl:ethylene glycol > ChCl:glycerol > ChCl:1,3-dimethylurea > ChCl:propylene glycol.

*Speed of sound.* There are only a few reports that describe the speed of sound for DESs, and the information available is mainly limited to deep eutectic solvents based on choline chloride. According to these reports, the speed of sound for DESs decreases with an increase in temperature, and in most cases linear dependence is observed [22,23]. However, in the case of DESs composed of glycerol and benzyltrimethylammonium chloride or benzyltributylammonium chloride, the temperature dependence of the speed of sound was found to be nonlinear, especially in the lower temperature region [29]. This type of behavior was also observed by Sanchez et al. for L-proline-based DESs [65].

## 3. Solubility of CO_2_ in Deep Eutectic Solvents

### 3.1. Experimental Methods for Measuring the Solubility of Carbon Dioxide in DESs

Over the years, many experimental methods have been developed to measure CO_2_ solubility in DESs. The most common ones are the isochoric saturation method and the gravimetric method. Additionally, the pressure drop method and the magnetic suspension balance method are used, but not as frequently. These methods enable taking measurements in a very wide range of temperatures and pressures. The majority of these methods are based on the assumption that the vapor pressure of DESs is negligible.

Isochoric saturation is the most common method used to measure the absorption of gases. In this method, a degassed DES is placed in a thermostated, well-sealed equilibrium cell at constant temperature, and then the equilibrium cell is evacuated, the gas from the thermostated reservoir is delivered to the cell, and the initial pressure is recorded. Next, the equilibrium is reached when the pressure in the system is constant [66,67,68,69,70,71,72,73].

The solubility is most often described by mole fraction and is derived by:

(1) $$x_{g}=\frac{n_{g}}{n_{g}+n_{DES}}$$

The solubility is determined from the difference between the initial gas pressure and the equilibrium gas pressure, and it is expressed as:

(2) $$n_{g}=n_{0}-n_{1}$$

where

(3) $$n_{\left(0,1\right)}=\frac{p_{\left(o,1\right)}V}{Z_{2}RT}$$

where *V* is the volume of the gas phase in the cell, *p*_0_ is the initial gas pressure, *p*_1_ is the equilibrium gas pressure, *R* is the universal gas constant, *T* is the temperature, and *Z*_2_ is the gas compressibility factor.

The apparatus used in the isochoric saturation method is relatively simple to design and can be used in a wide range of pressures. Unfortunately, during the absorption, the liquid volume may change. In order to overcome this obstacle, three approaches have been introduced: (a) the change in volume is assumed to be negligible, which was proven to be accurate for low gas pressures [66,74], (b) the volume expansion is measured using a cathetometer as a function of pressure [75], and (c) the percent volume expansion is correlated with the mole fraction of gas [74].

The gravimetric method is based on changes in the weight of the sample upon carbon dioxide absorption. In brief, the DES is placed in a thermostated absorption vial and then the gas is bubbled through the liquid at a known flow rate. The weight of the sample is recorded at regular time intervals. The equilibrium is obtained when the mass of the sample is constant [76,77,78,79,80]. This method is usually used for measurements at atmospheric pressure.

Isotherms are also measured with the magnetic suspension balance method [81,82]. This method is based on magnetic suspension coupling, which is responsible for the transmission of force from the measuring cell to the microbalance, and it allows for measurements in wide ranges of pressure and temperature.

Other methods, which are modification of those mentioned above, are also used [83].

#### 3.1.1. Impact of the Hydrogen Bond Acceptor on CO_2_ Capacity

In Table 2, the solubility of carbon dioxide in deep eutectic solvents is presented. One of the most important structural properties of a DES is the size of the cation in its salt which acts as the HBA. Deng et al. [69] examined the solubility of CO_2_ in levulinic acid-based DESs. They found that the solubilities in TEAC- and TEAB-based DESs were much lower than those observed for TBAC- and TBAB-based ones, which indicates that salts with a larger cation possess higher carbon dioxide absorption capacity than those with a smaller cation. Such behavior is the effect of the higher free volume of the sorbent at longer chain lengths [84]. Additionally, the effect of the anion is modest, as exchanging the chloride anion with the bromide anion results in a slight increase in carbon dioxide solubility. These conclusions are in compliance with the results obtained for DESs based on other HBAs, such as TBAB:AC (1:2) ≈ TBAC:AC (1:2) > TEAC:AC (1:2) [38]; TOAB:DecA (1:2) ≈ TOAC:DecA (1:2) > TBAC:DecA (1:2) [85]; and TBAC:LA (1:2) > TEAC:LA (1:2) > TMAC:LA (1:2) [82]. Furthermore, the effect of the free volume can be seen in tetrabutylammonium bromide- and in choline chloride-based DESs. TBAB-based DESs have more free volume, and thus the absorption capacity is higher than that of ChCl-based DESs [86].

Another important factor is the symmetry of the salt. Sarmad et al. [38] concluded that exchanging one ethyl group in TEAC:AC (1:2) for one benzyl group in BTEA:AC (1:2) results in lower carbon dioxide absorption capacity. Furthermore, introducing methyltrioctylammonium bromide/chloride instead of tetraoctylammonium bromide/chloride in decanoic acid-based DESs leads to lower solubility of carbon dioxide [85].

The last important factor is the chemical nature of the hydrogen bond acceptor. Introducing HBAs with groups that can interact with CO_2_ may improve its solubility. Liu et al. [87] proved that acetylcholine chloride-based DESs have higher carbon dioxide absorption capacity than choline chloride-based DESs, because of the ester group in ACC which has better affinity towards CO_2_ than the hydroxyl group in ChCl. The results obtained by Altamash et al. [88] showed that CO_2_ solubility in betaine-based DESs is higher than it is in alanine-based DESs due to the stronger interaction with the COO^−^ group than with the COOH.

**Table 2:** Solubility of carbon dioxide in deep eutectic solvents.

Lp.	HBA	HBD	Molar Ratio	T/K	P/bar	g_CO2_/g_DES_	Ref.
1	[bmim][MeSO_3_]	Urea	1:1	303.15	2.8–7.0	0.0071–0.0186	[89] ^1^
2	[HDBU][Im]	Ethylene glycol	7:3	313.15	1.0	0.141	[90] ^4^
3	[HDBU][Im]	Ethylene glycol	6:4	313.15	1.0	0.118	[90] ^4^
4	[HDBU][Im]	Ethylene glycol	5:5	313.15	1.0	0.109	[90] ^4^
5	[HDBU][Im]	Ethylene glycol	4:6	313.15	1.0	0.082	[90] ^4^
6	[HDBU][Im]	Ethylene glycol	3:7	313.15	1.0	0.063	[90] ^4^
7	[HDBU][Ind]	Ethylene glycol	7:3	313.15	1.0	0.117	[90] ^4^
8	[HDBU][Triz]	Ethylene glycol	7:3	313.15	1.0	0.108	[90] ^4^
9	[N_2222_][Im]	Ethylene glycol	1:2	298.15	1 atm	0.114	[91] ^4^
10	[N_2222_][Triz]	Ethylene glycol	1:2	298.15	1 atm	0.111	[91] ^4^
11	[P_2222_][Im]	Ethylene glycol	1:2	298.15	1 atm	0.106	[91] ^4^
12	[P_2222_][Triz]	Ethylene glycol	1:2	298.15	1 atm	0.106	[91] ^4^
13	1-Methylimidazolium hydrochloride	3-Amino-1-propanol	1:1	r.t.	1 atm	0.020	[92] ^4^
14	1-Methylimidazolium hydrochloride	3-Amino-1-propanol	1:2	r.t.	1 atm	0.095	[92] ^4^
15	1-Methylimidazolium hydrochloride	3-Amino-1-propanol	1:3	r.t.	1 atm	0.139	[92] ^4^
16	1-Methylimidazolium hydrochloride	3-Amino-1-propanol	1:4	r.t.	1 atm	0.194	[92] ^4^
17	1-Methylimidazolium hydrochloride	Diethylenetriamine	1:4	r.t.	1 atm	0.228	[92] ^4^
18	1-Methylimidazolium hydrochloride	Ethylenediamine	1:1	r.t.	1 atm	0.090	[92] ^4^
19	1-Methylimidazolium hydrochloride	Ethylenediamine	1:2	r.t.	1 atm	0.250	[92] ^4^
20	1-Methylimidazolium hydrochloride	Ethylenediamine	1:3	r.t.	1 atm	0.267	[92] ^4^
21	1-Methylimidazolium hydrochloride	Ethylenediamine	1:4	r.t.	1 atm	0.308	[92] ^4^
22	1-Methylimidazolium hydrochloride	Pentaethylenehexamine	1:4	r.t.	1 atm	0.084	[92] ^4^
23	1-Methylimidazolium hydrochloride	Tetraethylenepentamine	1:4	r.t.	1 atm	0.099	[92] ^4^
24	Acetylcholine chloride	1,2,4-Triazole	1:1	303.15	6.4–58.8	0.0012–0.0096	[93] ^1^
25	Acetylcholine chloride	Guaiacol	1:3	303.15	5.4–53.1	0.0007–0.0069	[87] ^1^
26	Acetylcholine chloride	Guaiacol	1:4	303.15	5.5–55.9	0.0007–0.0076	[87] ^1^
27	Acetylcholine chloride	Guaiacol	1:5	303.15	5.2–52.8	0.0008–0.0076	[87] ^1^
28	Acetylcholine chloride	Imidazole	2:3	303.15	3.0–57.3	0.0003–0.0100	[93] ^1^
29	Acetylcholine chloride	Imidazole	1:2	303.15	2.7–57.8	0.0008–0.0115	[93] ^1^
30	Acetylcholine chloride	Imidazole	1:3	303.15	5.3–56.8	0.0012–0.0129	[93] ^1^
31	Acetylcholine chloride	Levulinic acid	1:3	303.15	0.6–5.4	0.0019–0.0132	[69] ^1^
32	Alanine	Lactic acid	1:1	298.15	0.06–49.9	0.0013–0.1891	[88] ^2^
33	Alanine	Malic acid	1:1	298.15	0.06–49.9	0.0035–0.1823	[88] ^2^
34	Allyltriphenylphosphonium bromide	Diethylene glycol	1:4	303.15	1.6–19.4	0.0100–0.3207	[94] ^3^
35	Allyltriphenylphosphonium bromide	Diethylene glycol	1:10	303.15	1.6–19.5	0.0098–0.2547	[94] ^3^
36	Allyltriphenylphosphonium bromide	Diethylene glycol	1:16	303.15	1.7–19.6	0.0085–0.1404	[94] ^3^
37	Allyltriphenylphosphonium bromide	Phenol	1:4	313.15	2.2–13.3	0.0090–0.0786	[95] ^3^
38	Allyltriphenylphosphonium bromide	Phenol	1:6	313.15	1.8–13.2	0.0082–0.0787	[95] ^3^
39	Allyltriphenylphosphonium bromide	Triethylene glycol	1:4	303.15	1.4–19.5	0.0084–0.2826	[94] ^3^
40	Allyltriphenylphosphonium bromide	Triethylene glycol	1:10	303.15	1.5–19.5	0.0078–0.2101	[94] ^3^
41	Allyltriphenylphosphonium bromide	Triethylene glycol	1:16	303.15	1.4–19.6	0.0063–0.1887	[94] ^3^
42	Benzyltriethylammonium chloride	2-Ethylaminoethanol	1:4	303.15	10.2	0.090	[96] ^1^
43	Benzyltriethylammonium chloride	2-Methylaminoethanol	1:4	303.15	10.1	0.100	[96] ^1^
44	Benzyltriethylammonium chloride	Acetic acid	1:2	298.15	3.2–20.5	0.0056–0.0429	[38] ^1^
45	Benzyltrimethylammonium chloride	Acetic acid	1:2	298.15	2.2–20.4	0.0034–0.0640	[38] ^1^
46	Benzyltrimethylammonium chloride	Glycerol	1:2	298.15	3.9–20.3	0.0016–0.0114	[38] ^1^
47	Benzyltrimethylammonium chloride	Glycerol–H_2_O	1:2:0.05	298.15	2.1–20.2	0.0019–0.0125	[38] ^1^
48	Benzyltrimethylammonium chloride	Glycerol–H_2_O	1:2:0.11	298.15	2.6–20.3	0.0007–0.0143	[38] ^1^
49	Benzyltriphenylphosphonium chloride	Glycerol	1:12	298.15	10.0	0.0206	[97] ^1^
50	Betaine	Lactic acid	1:1	298.15	0.06–49.9	0.0022–0.1875	[88] ^2^
51	Betaine	Malic acid	1:1	318.15	0.05–49.9	0.0009–0.1536	[88] ^2^
52	BHDE^a^	Acetic acid	1:2	298.15	2.1–20.3	0.0028–0.0371	[38] ^1^
53	BHDE^a^	Glycerol–H_2_O	1:3:0.11	298.15	2.3–20.2	0.0016–0.0091	[38] ^1^
54	BHDE^a^	Lactic acid	1:2	298.15	2.8–20.9	0.0007–0.0219	[38] ^1^
55	Choline chloride	1,2-Propanediol	1:3	298.15	1.1–5.1	0.0001–0.0007	[68] ^1^
56	Choline chloride	1,2-Propanediol	1:4	298.15	1.0–5.0	0.0001–0.0007	[68] ^1^
57	Choline chloride	1,4-Butanediol	1:3	298.15	1.1–5.1	0.0001–0.0007	[68] ^1^
58	Choline chloride	1,4-Butanediol	1:4	298.15	1.1–5.1	0.0001–0.0007	[68] ^1^
59	Choline chloride	2,3-Butanediol	1:3	298.15	1.1–5.1	0.0001–0.0067	[68] ^1^
60	Choline chloride	2,3-Butanediol	1:4	298.15	1.1–5.1	0.0002–0.0008	[68] ^1^
61	Choline chloride	Cardanol	1:3	293.15	1.0	0.0037	[98] ^4^
62	Choline chloride	Cardanol	1:4	293.15	1.0	0.0038	[98] ^4^
63	Choline chloride	Cardanol	1:5	293.15	1.0	0.0039	[98] ^4^
64	Choline chloride	Diethanol amine	1:6	303.15	5.2–9.7	0.0133–0.0396	[86] ^1^
65	Choline chloride	Diethanolamine	1:6	298.15	10.0	0.0408	[97] ^1^
66	Choline chloride	Diethanolamine	1:12	r.t.	1 atm	0.196	[99] ^4^
67	Choline chloride	Diethylene glycol	1:3	303.15	5.6–11.1	0.0071–0.0140	[86] ^1^
68	Choline chloride	Diethylene glycol	1:4	303.15	5.2–11.2	0.0067–0.0146	[86] ^1^
69	Choline chloride	Diethylene glycol	1:3	298.15	11.3–51.3	0.0014–0.0074	[66] ^1^
70	Choline chloride	Diethylene glycol	1:4	298.15	11.0–50.9	0.0015–0.0082	[66] ^1^
71	Choline chloride	Ethanolamine	1:6	298.15	10.0	0.0749	[97] ^1^
72	Choline chloride	Ethanolamine	1:7	298.15	1.8–20.2	0.0359–0.2441	[100] ^1^
73	Choline chloride	Ethanolamine	1:7	298.15	1.8–20.4	0.0345–0.1577	[38] ^1^
74	Choline chloride	Ethanolamine	1:6	r.t.	1 atm	0.292	[99] ^4^
75	Choline chloride	Ethanolamine/aminoethylpiperazine	1:7:1	298.15	1.4–20.1	0.0256–0.2093	[100] ^1^
76	Choline chloride	Ethanolamine/diethanolamine	1:7:1	298.15	1.1–20.1	0.0188–0.1708	[100] ^1^
77	Choline chloride	Ethanolamine/methyldiethanolamine	1:7:1	298.15	1.8–20.1	0.0317–0.2020	[100] ^1^
78	Choline chloride	Ethanolamine/methyldiethanolamine	1:7:5	298.15	1.4–20.1	0.0205–0.1629	[100] ^1^
79	Choline chloride	Ethanolamine/piperazine	1:7:1	298.15	1.6–22.4	0.0409–0.3291	[100] ^1^
80	Choline chloride	Ethylene glycol	1:4	298.15	10.0	0.0133	[97] ^1^
81	Choline chloride	Ethylene glycol	1:8	298.15	10.0	0.0168	[97] ^1^
82	Choline chloride	Ethylene glycol	1:2	303.15	6.4–12.5	0.0135–0.0274	[86] ^1^
83	Choline chloride	Furfuryl alcohol	1:3	303.15	8.1–58.3	0.0012–0.0082	[27] ^1^
84	Choline chloride	Furfuryl alcohol	1:4	303.15	8.2–58.2	0.0013–0.0097	[27] ^1^
85	Choline chloride	Furfuryl alcohol	1:5	303.15	7.0–57.7	0.0013–0.0100	[27] ^1^
86	Choline chloride	Glycerol	1:3	298.15	10.0	0.0201	[97] ^1^
87	Choline chloride	Glycerol	1:8	298.15	10.0	0.0143	[97] ^1^
88	Choline chloride	Glycerol/acetic acid	1:1:1	298.15	2.6–20.1	0.0023–0.0191	[38] ^1^
89	Choline chloride	Glycerol/DBN	1:2:6	r.t.	1 atm	0.103	[101] ^4^
90	Choline chloride	Glycerol/DBN	1:2:3	r.t.	1 atm	0.096	[101] ^4^
91	Choline chloride	Glycerol/DBN	1:2:7	r.t.	1 atm	0.105	[101] ^4^
92	Choline chloride	Glycerol/DBN	1:2:8	r.t.	1 atm	0.103	[101] ^4^
93	Choline chloride	Glycerol/DBN	1:3:10	r.t.	1 atm	0.104	[101] ^4^
94	Choline chloride	Glycerol/DBU	1:2:6	r.t.	1 atm	0.036	[101] ^4^
95	Choline chloride	Glycerol/MTBD	1:2:6	r.t.	1 atm	0.100	[101] ^4^
96	Choline chloride	Guaiacol	1:3	303.15	5.5–55.3	0.0007–0.0062	[87] ^1^
97	Choline chloride	Guaiacol	1:4	303.15	10.0–54.9	0.0012–0.0068	[87] ^1^
98	Choline chloride	Guaiacol	1:5	303.15	4.7–53.9	0.0006–0.0071	[87] ^1^
99	Choline chloride	Guaiacol	1:3	293.15	1.0	0.0014	[98] ^4^
100	Choline chloride	Guaiacol	1:4	293.15	1.0	0.0015	[98] ^4^
101	Choline chloride	Guaiacol	1:5	293.15	1.0	0.0015	[98] ^4^
102	Choline chloride	Levulinic acid	1:3	303.15	7.9–57.0	0.0015–0.0112	[27] ^1^
103	Choline chloride	Levulinic acid	1:4	303.15	7.2–57.5	0.0014–0.0119	[27] ^1^
104	Choline chloride	Levulinic acid	1:5	303.15	7.1–56.7	0.0015–0.0126	[27] ^1^
105	Choline chloride	Methyldiethanol amine	1:6	303.15	4.4–11.0	0.0428–0.0665	[86] ^1^
106	Choline chloride	Methyldiethanol amine	1:7	303.15	5.9–10.3	0.0488–0.0896	[86] ^1^
107	Choline chloride	Phenol	1:2	298.15	9.9–49.9	0.0015–0.0086	[66] ^1^
108	Choline chloride	Phenol	1:3	298.15	10.4–50.8	0.0018–0.0090	[66] ^1^
109	Choline chloride	Phenol	1:4	298.15	10.8–50.9	0.0018–0.0093	[66] ^1^
110	Choline chloride	Triethanolamine	1:3	r.t.	1 atm	0.080	[99] ^4^
111	Choline chloride	Triethylene glycol	1:4	298.15	10.0	0.0130	[97] ^1^
112	Choline chloride	Triethylene glycol	1:3	298.15	10.9–50.4	0.0016–0.0084	[66] ^1^
113	Choline chloride	Triethylene glycol	1:4	298.15	11.9–51.4	0.0018–0.0085	[66] ^1^
114	Choline chloride	Urea	1:4	298.15	10.0	0.0142	[97] ^1^
115	Choline chloride	Urea	1:2.5	298.15	10.0	0.0114	[97] ^1^
116	Choline chloride	Urea	1:1.5	313.15	0.1–2.0	0.0003–0.0048	[70] ^1^
117	Choline chloride	Urea	1:2	313.15	0.1–2.0	0.0005–0.0080	[70] ^1^
118	Choline chloride	Urea	1:2.5	313.15	0.1–2.0	0.0003–0.0049	[70] ^1^
119	DBN	DMLU	1:2	318.15	1.0	0.0427	[102] ^4^
120	DBN	DMU	1:2	318.15	1.0	0.1734	[102] ^4^
121	DBN	EU	1:2	318.15	1.0	0.2302	[102] ^4^
122	DBN	EU	1:3	318.15	1.0	0.1931	[102] ^4^
123	Diethylamine hydrochloride	Guaiacol	1:3	303.15	5.4–51.4	0.0009–0.0081	[87] ^1^
124	Diethylamine hydrochloride	Guaiacol	1:4	303.15	6.2–52.5	0.0010–0.0086	[87] ^1^
125	Diethylamine hydrochloride	Guaiacol	1:5	303.15	5.7–52.0	0.0010–0.0088	[87] ^1^
126	Diethylenetriamine hydrochloride	3-Amino-1-propanol	1:4	r.t.	1 atm	0.183	[99] ^4^
127	Diethylenetriamine hydrochloride	Ethylenediamine	1:4	r.t.	1 atm	0.322	[99] ^4^
128	Diethylenetriamine hydrochloride	Tetraethylenepentamine	1:4	r.t.	1 atm	0.099	[99] ^4^
129	Ethanolamine hydrochloride	3-Amino-1-propanol	1:1	r.t.	1 atm	0.158	[92] ^4^
130	Ethanolamine hydrochloride	3-Amino-1-propanol	1:2	r.t.	1 atm	0.210	[92] ^4^
131	Ethanolamine hydrochloride	3-Amino-1-propanol	1:3	r.t.	1 atm	0.243	[92] ^4^
132	Ethanolamine hydrochloride	3-Amino-1-propanol	1:4	r.t.	1 atm	0.263	[92] ^4^
133	Ethanolamine hydrochloride	Diethylenetriamine	1:1	313.15	8.0	0.1132	[103] ^3^
134	Ethanolamine hydrochloride	Diethylenetriamine	1:3	313.15	8.0	0.1756	[103] ^3^
135	Ethanolamine hydrochloride	Diethylenetriamine	1:6	313.15	8.0	0.2412	[103] ^3^
136	Ethanolamine hydrochloride	Diethylenetriamine	1:9	313.15	8.0	0.2835	[103] ^3^
137	Ethanolamine hydrochloride	Diethylenetriamine	1:4	r.t.	1 atm	0.255	[92] ^4^
138	Ethanolamine hydrochloride	Ethylenediamine	1:1	313.15	8.0	0.1123	[103] ^3^
139	Ethanolamine hydrochloride	Ethylenediamine	1:3	313.15	8.0	0.1833	[103] ^3^
140	Ethanolamine hydrochloride	Ethylenediamine	1:6	313.15	8.0	0.3299	[103] ^3^
141	Ethanolamine hydrochloride	Ethylenediamine	1:9	313.15	8.0	0.3458	[103] ^3^
142	Ethanolamine hydrochloride	Ethylenediamine	1:1	r.t.	1 atm	0.235	[92] ^4^
143	Ethanolamine hydrochloride	Ethylenediamine	1:2	r.t.	1 atm	0.309	[92] ^4^
144	Ethanolamine hydrochloride	Ethylenediamine	1:3	r.t.	1 atm	0.365	[92] ^4^
145	Ethanolamine hydrochloride	Ethylenediamine	1:4	r.t.	1 atm	0.390	[92] ^4^
146	Ethanolamine hydrochloride	Monoethanolamine	1:1	313.15	8.0	0.0910	[103] ^3^
147	Ethanolamine hydrochloride	Monoethanolamine	1:3	313.15	8.0	0.1085	[103] ^3^
148	Ethanolamine hydrochloride	Monoethanolamine	1:6	313.15	8.0	0.1487	[103] ^3^
149	Ethanolamine hydrochloride	Monoethanolamine	1:9	313.15	8.0	0.1863	[103] ^3^
150	Ethanolamine hydrochloride	Pentaethylenehexamine	1:4	r.t.	1 atm	0.127	[92] ^4^
151	Ethanolamine hydrochloride	Tetraethylenepentamine	1:1	313.15	8.0	0.0835	[103] ^3^
152	Ethanolamine hydrochloride	Tetraethylenepentamine	1:3	313.15	8.0	0.1011	[103] ^3^
153	Ethanolamine hydrochloride	Tetraethylenepentamine	1:6	313.15	8.0	0.1484	[103] ^3^
154	Ethanolamine hydrochloride	Tetraethylenepentamine	1:9	313.15	8.0	0.1715	[103] ^3^
155	Ethanolamine hydrochloride	Tetraethylenepentamine	1:4	r.t.	1 atm	0.166	[92] ^4^
156	Ethanolamine hydrochloride	Triethylenetetramine	1:1	313.15	8.0	0.0982	[103] ^3^
157	Ethanolamine hydrochloride	Triethylenetetramine	1:3	313.15	8.0	0.1655	[103] ^3^
158	Ethanolamine hydrochloride	Triethylenetetramine	1:6	313.15	8.0	0.1765	[103] ^3^
159	Ethanolamine hydrochloride	Triethylenetetramine	1:9	313.15	8.0	0.2045	[103] ^3^
160	Guanidinium hydrochloride	Ethanolamine	1:2	298.15	2.3–20.2	0.0135–0.0732	[38] ^1^
161	L-arginine	Glycerol	1:5	353.15	1 atm	0.1677	[104] ^4^
162	L-arginine	Glycerol	1:6	353.15	1 atm	0.1937	[104] ^4^
163	L-arginine	Glycerol	1:7	353.15	1 atm	0.1939	[104] ^4^
164	Methyltrioctylammonium bromide	Decanoic acid	1:2	298.15	0.9–19.9	0.0033–0.0783	[85] ^2^
165	Methyltrioctylammonium chloride	Decanoic acid	1:2	298.15	0.9–19.9	0.0024–0.0595	[85] ^2^
166	Methyltriphenylphosphonium bromide	1,2-Propanediol	1:4	298.15	2.2–20.3	0.0010–0.0242	[38] ^1^
167	Methyltriphenylphosphonium bromide	Acetic acid	1:4	298.15	1.7–20.1	0.0032–0.1330	[38] ^1^
168	Methyltriphenylphosphonium bromide	Ethanolamine	1:6	298.15	10.0	0.0716	[97] ^1^
169	Methyltriphenylphosphonium bromide	Ethanolamine	1:7	298.15	10.0	0.0643	[97] ^1^
170	Methyltriphenylphosphonium bromide	Ethanolamine	1:8	298.15	10.0	0.0632	[97] ^1^
171	Methyltriphenylphosphonium bromide	Ethylene glycol	1:2	298.15	1.9–20.2	0.0020–0.0155	[38] ^1^
172	Methyltriphenylphosphonium bromide	Glycerol	1:4	298.15	1.6–20.3	0.0004–0.0127	[38] ^1^
173	Methyltriphenylphosphonium bromide	Levulinic acid	1:3	298.15	3.0–2.1	0.0011–0.0303	[38] ^1^
174	Methyltriphenylphosphonium bromide	Levulinic acid/acetic acid	1:3:0.03	298.15	2.9–20.6	0.0077–0.0579	[38] ^1^
175	Monoethanolamine hydrochloride	Ethylenediamine	1:1	r.t.	1 atm	0.205	[105] ^4^
176	Monoethanolamine hydrochloride	Ethylenediamine	1:2	r.t.	1 atm	0.244	[105] ^4^
177	Monoethanolamine hydrochloride	Ethylenediamine	1:3	r.t.	1 atm	0.315	[105] ^4^
178	Monoethanolamine hydrochloride	Ethylenediamine	1:4	r.t.	1 atm	0.308	[105] ^4^
179	n-Butyltriphenylphosphonium bromide	Ethylene glycol	1:12	298.15	10.0	0.0201	[97] ^1^
180	Tetrabutylammonium bromide	2-Ethylaminoethanol	1:4	303.15	10.0	0.071	[96] ^1^
181	Tetrabutylammonium bromide	2-Methylaminoethanol	1:4	303.15	10.5	0.106	[96] ^1^
182	Tetrabutylammonium bromide	3-Amino-1-propanol	1:2	r.t.	1 atm	0.111	[92] ^4^
183	Tetrabutylammonium bromide	3-Amino-1-propanol	1:3	r.t.	1 atm	0.156	[92] ^4^
184	Tetrabutylammonium bromide	3-Amino-1-propanol	1:4	r.t.	1 atm	0.181	[92] ^4^
185	Tetrabutylammonium bromide	Acetic acid	1:2	298.15	3.9–20.1	0.0060–0.0497	[38] ^1^
186	Tetrabutylammonium bromide	Aminomethylpropanol	1:3	r.t.	1 atm	0.105	[92] ^4^
187	Tetrabutylammonium bromide	Aminomethylpropanol	1:4	r.t.	1 atm	0.122	[92] ^4^
188	Tetrabutylammonium bromide	Diethanol amine	1:6	303.15	6.1–10.1	0.0157–0.0367	[86] ^1^
189	Tetrabutylammonium bromide	Diethanolamine	1:6	298.15	10.0	0.0373	[97] ^1^
190	Tetrabutylammonium bromide	Diethanolamine	1:2	r.t.	1 atm	0.096	[99] ^4^
191	Tetrabutylammonium bromide	Diethylene glycol	1:2	303.15	7.2–13.9	0.0086–0.0272	[86] ^1^
192	Tetrabutylammonium bromide	Diethylene glycol	1:3	303.15	6.9–12.0	0.0113–0.0271	[86] ^1^
193	Tetrabutylammonium bromide	Diethylene glycol	1:4	303.15	5.9–10.5	0.0146–0.0290	[86] ^1^
194	Tetrabutylammonium bromide	Ethanolamine	1:6	298.15	10.0	0.0591	[97] ^1^
195	Tetrabutylammonium bromide	Ethanolamine	1:6	298.15	3.5–20.2	0.0193–0.1223	[38] ^1^
196	Tetrabutylammonium bromide	Ethanolamine	1:7	298.15	3.8–20.4	0.0235–0.1324	[38] ^1^
197	Tetrabutylammonium bromide	Ethanolamine	1:5	r.t.	1 atm	0.197	[99] ^4^
198	Tetrabutylammonium bromide	Ethylene glycol	1:2	303.15	4.1–12.8	0.0045–0.0168	[86] ^1^
199	Tetrabutylammonium bromide	Ethylene glycol	1:3	303.15	5.0–12.5	0.0059–0.0189	[86] ^1^
200	Tetrabutylammonium bromide	Ethylene glycol	1:4	303.15	5.4–13.7	0.0074–0.0201	[86] ^1^
201	Tetrabutylammonium bromide	Levulinic acid	1:3	303.15	0.7–5.7	0.0015–0.0119	[69] ^1^
202	Tetrabutylammonium bromide	Methyldiethanol amine	1:3	303.15	4.2–10.0	0.0244–0.0663	[86] ^1^
203	Tetrabutylammonium bromide	Methyldiethanol amine	1:4	303.15	5.0–10.2	0.0340–0.0800	[86] ^1^
204	Tetrabutylammonium bromide	Triethanolamine	1:3	298.15	10.0	0.2070	[97] ^1^
205	Tetrabutylammonium bromide	Triethanolamine	1:2	r.t.	1 atm	0.025	[99] ^4^
206	Tetrabutylammonium chloride	Acetic acid	1:2	298.15	3.5–20.0	0.0081–0.0621	[38] ^1^
207	Tetrabutylammonium chloride	Decanoic acid	1:2	298.15	0.9–19.9	0.0027–0.0668	[85] ^2^
208	Tetrabutylammonium chloride	Lactic acid	1:2	308.15	0.9–19.9	0.0016–0.0420	[82] ^2^
209	Tetrabutylammonium chloride	Levulinic acid	1:3	303.15	0.6–5.6	0.0015–0.0133	[69] ^1^
210	Tetrabutylphosphonium bromide	Diethylene glycol	1:4	313.15	1.9–14.0	0.0070–0.0776	[95] ^3^
211	Tetrabutylphosphonium bromide	Phenol	1:4	313.15	2.3–15.8	0.0092–0.0792	[95] ^3^
212	Tetraethylammonium bromide	Levulinic acid	1:3	303.15	0.7–5.6	0.0013–0.0106	[69] ^1^
213	Tetraethylammonium chloride	Acetic acid	1:2	298.15	2.8–20.2	0.0063–0.0518	[38] ^1^
214	Tetraethylammonium chloride	Acetic acid	1:3	298.15	4.0–20.2	0.0193–0.1223	[38] ^1^
215	Tetraethylammonium chloride	Lactic acid	1:2	308.15	1.0–19.9	0.0012–0.0298	[82] ^2^
216	Tetraethylammonium chloride	Levulinic acid	1:3	303.15	0.7–5.6	0.0015–0.0121	[69] ^1^
217	Tetraethylammonium chloride	Octanoic acid	1:3	298.15	3.5–20.2	0.0069–0.0612	[38] ^1^
218	Tetramethylammonium chloride	Acetic acid	1:4	298.15	2.9–21.0	0.0053–0.0687	[38] ^1^
219	Tetramethylammonium chloride	Lactic acid	1:2	308.15	1.0–19.9	0.0011–0.0282	[82] ^2^
220	Tetraoctylammonium bromide	Decanoic acid	1:2	298.15	0.9–19.9	0.0023–0.0586	[85] ^2^
221	Tetraoctylammonium chloride	Decanoic acid	1:2	298.15	0.9–19.9	0.0023–0.0574	[85] ^2^
222	Tetraoctylammonium chloride	Decanoic acid	1:1.5	298.15	0.9–19.9	0.0027–0.0667	[85] ^2^
223	Tetrapropylammonium chloride	Acetic acid	1:6	298.15	3.5–20.3	0.0110–0.0757	[38] ^1^
224	Tetrapropylammonium chloride	Ethanolamine	1:4	298.15	4.8–20.1	0.0149–0.0628	[38] ^1^
225	Tetrapropylammonium chloride	Ethanolamine	1:7	298.15	3.6–20.2	0.0754–0.1551	[38] ^1^
226	Thioacetamide hydrochloride	Ethylenediamine	1:3	r.t.	1 atm	0.101	[105] ^4^
227	Triethanolamine hydrochloride	Ethylenediamine	1:3	r.t.	1 atm	0.175	[105] ^4^
228	Triethylmethylammonium chloride	Acetic acid	1:2	298.15	2.0–18.4	0.0036–0.0518	[38] ^1^
229	Triethylmethylammonium chloride	Ethylene glycol	1:2	298.15	1.4–1.3	0.0027–0.0276	[38] ^1^
230	Triethylmethylammonium chloride	Glycerol	1:2	298.15	1.5–16.5	0.0007–0.0191	[38] ^1^
231	Triethylmethylammonium chloride	Glycerol–H_2_O	1:2:0.05	298.15	2.3–19.8	0.0004–0.0289	[38] ^1^
232	Triethylmethylammonium chloride	Glycerol–H_2_O	1:2:0.11	298.15	1.4–17.4	0.0011–0.0292	[38] ^1^
233	Triethylmethylammonium chloride	Lactic acid	1:2	298.15	1.4–18.6	0.0021–0.0234	[38] ^1^
234	Triethylmethylammonium chloride	Levulinic acid	1:2	298.15	1.4–16.2	0.0025–0.0270	[38] ^1^
235	Trimethylglycine	Glycolic	1:2	298.15	10.0	0.00764	[106] ^4^
236	Trimethylglycine	Oxalic acid dihydrate	1:2	298.15	10.0	0.00048	[106] ^4^
237	Trimethylglycine	Phenylacetic acid	1:2	298.15	10.0	0.00992	[106] ^4^
238	Urea hydrochloride	Ethylenediamine	1:3	r.t.	1 atm	0.117	[105] ^4^

^1^ Isochoric saturation method; ^2^ magnetic suspension balance; ^3^ pressure drop method; ^4^ gravimetric method.

#### 3.1.2. Effect of Hydrogen Bond Donor

**Figure 3:**
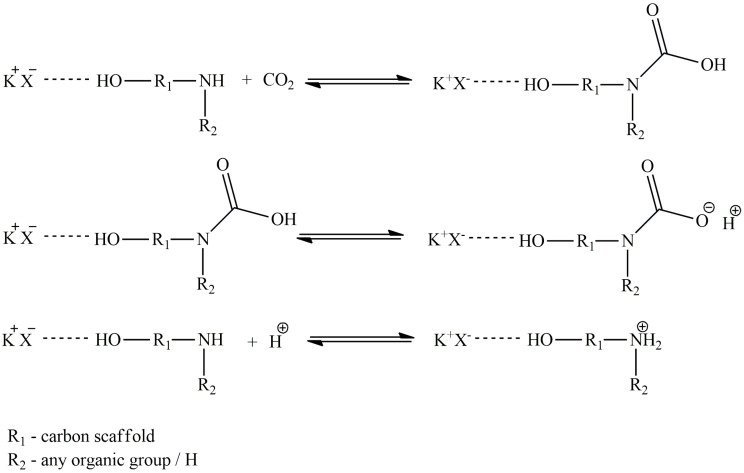
Mechanism of CO_2_ absorption in DESs based on primary and secondary amines.

The hydrogen bond donor determines the nature of interactions between the DES and carbon dioxide, either chemical or physical. The use of amines and alkanolamines as HBDs results in a chemical reaction between the DES and CO_2_, i.e., chemical absorption occurs (as presented in Figure 3), while other HBDs such as amides, glycols, sugars, and acids are responsible for physical absorption.

The early work of Chen et al. [68] helped to elucidate the effect of the position of hydroxyl groups on the physical absorption in choline chloride-based DESs. The results showed that higher absorption is observed for dihydric alcohols with hydroxyl groups located closer to each other in carbon scutter (2,3-butanediol) than for dihydric alcohols with hydroxyl groups placed further apart (1,4-butanediol). Ghaedi et al. [94] examined the impact of the ether groups and alkyl chain length of the HBD on DESs based on allyltriphenylphosphonium bromide (ATPPB) and diethylene glycol or triethylene glycol (TEG). At 303.15 K and 1.95 MPa, the solubility of CO_2_ was higher for ATPPB:TEG DESs (1:4) (x_CO2_ = 0.5583) than for ATPPB:DEG DESs (1:4) (x_CO2_ = 0.5407). The additional ethylene group and higher free volume contributes to higher CO_2_ solubility for TEG DESs [107]; hence, the additional ether group in TEG also improves the absorption capacity [108]. Similar behavior was observed for deep eutectic solvents based on TBAB and glycol or ethylene glycol [86]. The mole fraction of CO_2_ for TBAB:EG (1:2) at 303.15 K and 1 MPa was 0.0429, while for TBAB:DEG (1:2) in the same conditions it was 0.0593. Lu et al. [27] conducted a study on the solubility of carbon dioxide in the eutectic mixture of levulinic acid or furfuryl alcohol and choline chloride. They concluded that solubility is higher for the levulinic acid-based DESs than for the furfuryl alcohol-based DESs, which they attributed to the higher affinity of the –COOH group towards CO_2_ than that of the -OH group. These authors reported that the strength of the interactions between functional groups in the HBDs and carbon dioxide rises in the following order: amide > carbonyl group > ether bond > hydroxyl group. The only exceptions are glycerol-based DESs, for which CO_2_ solubility is higher than for DESs based on HBD with a carbonyl group. Additionally, intramolecular interactions may play a key role in carbon dioxide absorption capacity. For triethylmethylammonium chloride-based DESs, the solubility follows the sequence TEMA:LA < TEMA:LV < TEMA:AC [38]. The poorest solubility, in lactic acid-based DESs, may result from the proximity of the carboxyl group and the hydroxyl group, which gives stronger intermolecular bonding than in levulinic acid or acetic acid. Consequently, the bonding is more difficult to break.

Alkanolamines and amines may be suitable components of DESs for carbon dioxide absorption. They absorb the gas chemically, which leads to higher carbon dioxide solubility than for other DESs. The solubility can be even higher than in the commonly used 30% wt. solution of monoethanolamine [97]. In general, the structural properties of alkanolamines and amines show a similar impact on their carbon dioxide absorption capacity as in other HBDs. For example, Pishro et al. [103] examined the solubility of CO_2_ in triethylenetetramine (TETA), diethylenetriamine (DETA), and tetraethylenepentamine (TEPA). Taking into account the same components’ molar ratios, the solubility was found to be the highest for EAHC:TETA (1:6), and slightly lower for EAHC:DETA (1:6), whereas the lowest solubility was observed for EAHC:TEPA (1:6). This led to the conclusion that carbon dioxide solubility is higher for DESs based on HBDs with a longer alkyl chain and with more amine groups in their structure. The influence of the alkyl chain length on carbon dioxide capacity might be different for DESs based on alkanolamines with only secondary amine groups. Haider et al. [96] conducted research on CO_2_ solubility for 2-methylaminoethanol- and 2-ethylaminoethanol-based DESs. The absorption capacity of CO_2_ for TBAB-MAE (1:4) was 0.30 mol_CO2_/mol_DES_, while for TBAB-EAE (1:4) it was 0.22 mol_CO2_/mol_DES_ in the same conditions. This phenomenon was attributed to a lower steric hindrance caused by a shorter alkyl chain in the MAE-based DESs. The other important factor is the substitution of amines. Trisubstituted amines do not form carbamic acid with CO_2_, and hence the absorption is simply physical, and the solubility of carbon dioxide is lower [86,109].

#### 3.1.3. Effect of HBA/HBD Molar Ratio

The molar ratio of the hydrogen bond acceptor to the hydrogen bond donor has various effects on carbon dioxide solubility in deep eutectic solvents. For DESs based on dihydric alcohols, the effect of the HBA/HBD molar ratio depends on the structure of HBD. For ChCl:2,3-butanediol, the solubility increases from 0.0308 mol·kg^−1^ for 1:3 molar ratio to 0.0382 mol·kg^−1^ for 1:4 molar ratio at 110 kPa and 293.15 K, respectively, while for ChCl:1,4-butanediol and ChCl:1,2-propanediol it decreases from 0.0330 mol·kg^−1^ to 0.0306 mol·kg^−1^ and from 0.0365 mol·kg^−1^ to 0.0355 mol·kg^−1^, respectively [68]. For ATPPB:DEG and ATPPB:TEG DESs, the solubility decreases with an increase in the amount of the HBDs from 1:4 to 1:16 in the DES, due to the decrease in the molar volume and the free volume [94]. Surprisingly, for ChCl:DEG and TBAB:DEG DESs, the increase in the HBA/HBD molar ratio from 1:3 to 1:4 enhances the solubility of carbon dioxide, which may be attributed to the weaker hydrogen bonds at higher molar ratios [66,86].

Considering phenol and its derivatives, the effect of the HBA/HBD molar ratio is also complex. For ChCl:phenol DESs, the solubility increases when the ratio changes from 1:2 to 1:4 [66]. The same behavior can be observed for ChCl:GC, DH:GC, and ACC:GC DESs [87]. Additionally, Pishro et al. [103] attributed the increase in carbon dioxide absorption capacity with an increasing HBA/HBD molar ratio for DESs based on a variety of different amines and alkanolamines acting as HBD to a drop in the DESs’ viscosity. Lower viscosity results in lower diffusion resistance and thus in increasing the solvent fluidity and, consequently, in improving the mass transfer. For ChCl:MEA DESs, the absorption of CO_2_ increases gradually when the molar ratio changes from 1:2 to 1:4, but a further increase in the molar ratio to 1:6 has no effect on carbon dioxide uptake [109]. Once more, this phenomenon might be explained with the hydrogen bonds’ net. At higher molar ratios, the absorption of CO_2_ changes and, as a result, ChCl and MEA cannot be sufficiently mixed to form hydrogen bonds. In their work, Ali et al. [97] revealed that changing the molar ratio of HBA/HBD in MTPB:MAE from 1:6 to 1:8 results in a decrease in solubility, which made these authors conclude that the formed DES possesses various properties that cannot be predicted by simply considering the contribution effect of its components.

#### 3.1.4. Synergistic Effect

Shukla and Mikkola [92] tried to combine solvatochromic polarity parameters, which probe intermolecular interactions, with carbon dioxide absorption capacity. These parameters include:

- Electronic transition energy (*E_T_*(30)) which stands for the hydrogen bond donor–acceptor forces, π–π interactions, and dipole–dipole interactions present in a solvent;
- Dipolarity/polarizability (π*) which is a measure of the electrolytic strength of the medium;
- Hydrogen bond donor acidity (α) which denotes the donating ability of the hydrogen bond donor;
- Hydrogen bond acceptor basicity (β) which denotes the strength of the solvent’s hydrogen bond acceptor.

Solvatochromic polarity parameters are determined via the UV-VIS spectra of solvents by using the appropriate dyes: Reichardt’s dye 30 is used for *E_T_*(30), and *N*,*N*-diethyl-4-nitroaniline for π*. The hydrogen bond acceptor basicity is defined by the spectroscopic shift of 4-nitroaniline with respect to *N*,*N*-diethyl-4-nitroaniline, while the hydrogen bond donor acidity can be calculated based on *E_T_*(30) and π*.

Shukla and Mikkola [92] investigated the influence of the HBA/HBD molar ratio on solvatochromic polarity parameters and CO_2_ absorption capacity. They did not find a clear relationship between *E_T_*(30) and π* for the various DESs. For deep eutectic solvents based on protic HBAs, the reverse relationship between the value of *E_T_*(30) and carbon dioxide absorption capacity was observed, which suggests the involvement of non-polar interactions in CO_2_ capture. Additionally, these researchers stated that the high carbon dioxide solubility can be attributed not to basicity but rather to the equilibrium between α and β. This so-called synergistic effect occurs when the |α − β| is equal or close to 0. Additionally, the standard enthalpy change and the standard entropy change should be positive and *E_T_*(30) should be low. In this case, there is no energy difference between the HBAs and HBDs in a DES, which means that both components make stable sites that interact with CO_2_ [110].

In their other work, Shukla and Mikkola [99] observed much lower solubilities of carbon dioxide in TBAB-based DESs than in ChCl-based DESs despite the similar HBD components. On comparison of α and β, it was noticed that the synergistic effect was higher for ChCl-based DESs, which was the reason for the higher solubility of this class of DESs. The experiment on TBAB:3-amino-1-propanol showed that the reverse relationship between the molar ratio of HBA/HBD and carbon dioxide capacity can be explained via the synergistic effect. Unfortunately, there was no clear relationship between the synergistic effect and the carbon dioxide absorption capacity in AP-based DESs with different HBAs. It was suggested that other factors, such as the free volume or the strength of hydrogen interactions between the components, should be taken into consideration [111].

## 4. Practical Issues of Carbon Dioxide Separation

Taking into account the valuable properties of DESs, their safety and environmental friendliness in comparison to other solvents, and the ease of their synthesis, these solvents could replace other media used for gas separation. Their carbon dioxide capture capacity has ensured that a number of very promising approaches are being considered. However, considerable and constant effort needs to be made in order to use these innovative solvents on a large scale.

From the technological point of view, DESs’ most valuable property is their low volatility, ensuring low solvent loss. In addition, the possibility of tuning DESs’ physicochemical properties by changing the mixtures’ composition and the structure of their components is a key issue in efficient process design. An important aspect of controlling the physicochemical properties of deep eutectic liquids is the addition of classical solvents. From the point of view of CO_2_ separation, water is of particular importance and can be used to modulate the physicochemical properties of the solvent, especially its mass transfer properties such as viscosity. The formation of a DES aqueous mixture not only changes the physical properties of the deep eutectic solvent but also has a very large influence on its interactions with carbon dioxide, mostly due to the formation of new bonds between H_2_O and the DES.

Research on DESs and water mixtures is primarily focused on the thermodynamic properties of the mixtures, and it mainly concerns DESs based on choline chloride as HBA. The effect of water content on the solubility of carbon dioxide in a DES has been investigated only in a few studies. The results for DESs based on ChCl showed that an increase in the water content results in a decrease in CO_2_ solubility [112,113]. However, the results obtained for amine-based DESs show the opposite behavior. Trivedi et al. reported that a small addition of water (up to 10%) to MEACl:EDA with a 1:3 molar ratio can improve carbon dioxide solubility, but further H_2_O addition leads to a reduction in CO_2_ solubility [105]. Li et al. reported similar results for TMAC:MEA and TEAC:MEA deep eutectic solvents, and they observed only a slight effect of water content on a ChCl:MEA DES [109].

Some possible mechanisms of water influence on carbon dioxide capacity can be found in the literature. Su et al. reported that a decrease in the solubility of carbon dioxide with increasing water content might occur due to a decrease in the concentration of effective reactants [112]. Shukla et al. hypothesized that water competes with CO_2_ for the active sites of DESs, which affects CO_2_ uptake [92]. The increased solubility of carbon dioxide might be a result of weakening intermolecular hydrogen bond interactions within the DES structure by the forming of new interactions between water and the DES that increase the free volume and, in consequence, decrease the solvent viscosity [105,109].

Nevertheless, the addition of water to DESs alters many physical properties of the solvents, but the most important from the point of view of gas separation technologies is a drop in DES viscosity. In the study performed by Ma et al. [114], the viscosity of BTMA-:GLY (1:2) DES dropped from 716 to 20 mPa·s at 0.11 mole fraction of water, while the solubility increased by 25%.

For a better understanding of how H_2_O interacts with DESs, a few theoretical studies based on molecular dynamics (MD) were conducted. MD simulations performed by Shah et al. allowed for exploration of interactions in ChCl:Urea (1:2) aqueous solutions [115]. It was shown that Cl^−^ becomes preferentially hydrated by water compared with urea or choline chloride. Additionally, the effect of water was divided into three ranges based on the mass fraction of H_2_O. In the first range (w_H2O_ < 5% wt.), the number of hydrogen bonds increases, reaching the maximum at w_H2O_ ≈ 2%. In the next range (from 5% to 25% wt.), choline chloride and urea molecules are hydrated by H_2_O, resulting in low diffusivity. In the last range (water mass fraction above 25%), the anions and urea show high diffusivity. Additionally, this simulation indicated that chloride anions interact more strongly with water molecules than with urea molecules.

ChCl:urea (1:2), ChCl:Gly (1:2), and ChCl:EG (1:2) water systems were also studied by Zhekenov et al. [116]. Molecular dynamics simulations they carried out showed similar behavior for different DESs. Below 5% wt., H_2_O molecules form H-bonds with ions and hydrogen bond donors, which leads to the absorption of water into the DES structure. Water content above 5% leads to drastic changes of DES properties by dampening the intramolecular interactions in the DES. First-principal MD simulations performed for a ChCl:urea (1:2) equimolar mixture with water showed that the addition of H_2_O breaks the strong H-bonds between the H atoms of urea and the chloride anion. This is due to preferentially hydrating Cl^−^ by water and forming urea–water H-bonds [117]. Studies of pure ChCl:urea (1:2) at high dilution showed that, even for high molar fractions of water (x_H2O_ = 0.9), the ions are only partially hydrated. Additionally, as the water content decreases, the ion-pairing of cation and anion becomes dominant over the hydration of ions, which leads to the conclusion that the non-ideal behavior is the result of competition between these two interactions [118].

Molecular dynamics is also a powerful tool to assess the technological feasibility of DES use in separation processes. It can reveal structural information that may be hard or impossible to obtain with other techniques. The first works in this field focused on explaining the mechanism behind the formation of DESs and the freezing point depression. It was proven that, in the case of choline chloride-based DESs, a strong hydrogen bond formed between the chloride anion and the hydroxyl group of the HBD is responsible for the unique behavior of DESs [119,120]. Similar behavior was reported for HBAs with a bromide anion [121]. Migliorati et al. proved that other groups in HBAs which are capable of forming hydrogen bonds have a very large impact on the hydrogen bond network in DESs [122]. They compared the structures of choline chloride- and benzyltrimethylammonium chloride-based DESs and revealed that the -OH group in ChCl forms additional hydrogen bonds with HBD molecules, which leads to the formation of a three-dimensional arrangement of all the species. This structure is very different from those formed in a benzyltrimethylammonium chloride-based DES. The researchers additionally found that the introduction of even a small functional group leads to a change in the balance among all the different forces. Ferreira et al. examined the structure of a ChCl:propylene glycol (PG) 1:2 DES [123]. From the radial distribution function (RDF), they found that the HBD molecule is placed between CH^+^ and Cl^−^. Additionally, PG is surrounded with anion molecules rather than with other PG or cation molecules. Furthermore, they observed that CL^−^–HBD interactions are the most common in this system, while HBD–HBD are the least common, which is the opposite of the case in the ChCl:EG (1:2) system [124]. In their study on 1,8-cinole (CN)-based DESs, Rozas et al. proved that not all HBDs interact with HBAs in the same way [125]. In the case of the CN:malic acid (MA) system, there is a negligible difference between the hydrogen bonds developed through the central and the terminal hydroxyl groups of the HBD. However, in the case of lactic acid (LA)-based DESs, the interactions of the COOH group were stronger than those of the OH group. The study on DESs containing methyltriphenylphosphonium bromide and monoethanolamine showed almost five times stronger interactions between Br^−^ and the hydroxyl group than with the amine group [126]. Pour et al. examined the influence of the HBA/HBD molar ratio on the strength of hydrogen bonds in ChCl:glucose deep eutectic solvents [127]. They observed a gradual decrease in the hydrogen bonds between the two species with an increase in glucose concentration. Alizadeh et al. conducted a study in which they estimated microheterogenity in choline chloride and some of its derivatives coupled with ethylene glycol in 1:2 molar ratio [128]. They investigated the influence of the alcohol-substituted side chain, the symmetry and length of the alkyl chains, and the number of hydroxyl groups. The researchers proved microheterogenity, in some cases even strong microheterogenity, in all the systems studied. They also observed that the polar groups always tended to form one domain, while the non-polar chains were highly dislocated for the compounds, with the side chain length lower than eight carbon units. However, when carbon units were eight or more, the non-polar groups also formed connected domains, indicating strong microheterogenity.

MD can be successfully used for predicting the dynamic, physicochemical, and structural properties of deep eutectic solvents [129,130,131]. With the use of MD, Kumar et al. examined the solubility of CO_2_, H_2_S, CH_4_, and N_2_ in DESs based on ethaline, reline, glycine, and oxaline, and they observed the highest solubility for H_2_S and the lowest for N_2_ [132]. Methyl diethanoloamine and choline chloride were investigated in the separation of H_2_S, CO_2_, and CH_4._ This study confirmed that H_2_S solubility was higher than that of CH_4_ [133]. However, MD needs further improvement, due to the poor estimation of transport properties, mostly via improvement of force fields, indicating that further work should be conducted [33].

To enhance CO_2_ absorption capacity, a co-solvent that absorbs this gas chemically can be used. Muthu et al. [134] conducted a study in which they determined carbon dioxide absorption capacity in a ChCl:U (1:2) DES mixed with various alkanolamines. It was concluded that CO_2_ uptake was higher in alkanolamine-DES systems than that observed for pure DES or aqueous solutions of alkanolamines. This conclusion is compatible with the results obtained by Sarmad et al. [100] for amine-functionalized ChCl:MAE (1:7) DES.

Additionally, in the study conducted by Li et al. [109], it was proven that the addition of inorganic salts to a DES can change its ability to absorb CO_2_. DESs containing NiCl_2_, FeCl_3_, CoCl_2_, or CuCl_2_ possess the same carbon dioxide absorption capacity as pure DESs, while the addition of ZnCl_2_, LiCl, or NH_4_Cl to DESs results in higher CO_2_ absorption. Ni^2+^, Fe^3+^, Co^2+^, and Cu^2+^ possess unpaired electrons on their electron shells, which facilitates their easy bonding with carbonyl oxygen.

For separation purposes, DESs have been used in several configurations. One of the approaches proposed by Lian et al. incorporated an amino acid-based DES (L-arginine:ethylene glycol in 1:5 molar ratio) into the PEBAX membrane at 5–20% g/g. With an increase in DES loading, CO_2_ permeability decreased, but the CO_2_/N_2_ selectivity increased up to 15% loading. For 15% DES loading, the selectivity increase reached 21% and the permeability loss was only 5% [135]. N_2_ permeability remained almost constant, however. Considering the temperature effect, it was found that CO_2_ and N_2_ permeability both increase with increasing temperature, while the selectivity decreases.

Saeed et al. examined a supported liquid membrane based on PVDF and a DES composed of betaine and urea, glycerol, or ethylene glycol in 1:3 molar ratio. They obtained a permeability of 31.23 for the glycerol-based DES and 35.67 barrers for the DES based on urea, having the separation factor CO_2_/CH_4_ of 43.32 and 57.53, respectively [136]. Potassium carbonate with glycerol or ethylene glycol was also investigated in a supported liquid membrane system, resulting in CO_2_ permeability of 34 and 20 barrers and CO_2_ selectivity over CH_4_ of 59 and 34, respectively [137].

Wu et al. calculated ideal selectivity for the H_2_S/CO_2_ system using a 1-ethyl-3-methylimidazolium chloride plus imidazole DES and obtaining the values of 30.9, 23.7, and 17.2, respectively, for 2:1; 1:1, and 1:2 ratios at 298.2 K [138]. An important technological parameter that is crucial for the economics of the process is the regenerability of the sorption media. The efficiency of the process and the operational parameters needed for the regeneration result from the strength of interactions between the DES and the gas, so the DES structure plays a crucial role, and it has been confirmed that the ratio of DES components affects the desorption process [139,140]. When physical interactions are observed, the regeneration is energy and time efficient.

In ten cycles of absorption and desorption, the capacity remained unchanged, thus showing the potential for a long-term use [138]. Cui et al. studied the reusability of [P_2222_][Triz]:EG (1:2) DESs. These authors claim that no discernible decrease in capacity was obtained, but from analyzing the data one can see the capacity decrease in five cycles of absorption and desorption [91].

## 5. Conclusions and Perspectives

Large amounts of air pollution with acid gases have driven the search for new sorption media and, to be in line with sustainable development goals, media that are environmentally friendly. This article summarizes the properties of DESs and their suitability for carbon dioxide separation. Deep eutectic solvents have attracted much attention due to their unique properties and low production cost. Their nature, especially their negligible vapor pressure and non-flammability, makes them useful and safe in gas separation processes. The impact of the hydrogen bond donor, hydrogen bond acceptor, and molar ratio need to be thoroughly analyzed to ensure high CO_2_ absorption capacity and, at the same time, high selectivity in regard to other components of the purified streams. Therefore, careful selection should be performed, and the development of molecular dynamics methods would make that easier, less time consuming, and economically advantageous. Considering their tunability, DESs have high potential in separation processes, as they can be designed to obtain desirable properties and capacity for a specific application. Still, the major challenge is their relatively high viscosity. It results from the presence of hydrogen bonds and electrostatic and van der Waals interactions between the individual components of the DESs. However, even a small addition of water can significantly decrease viscosity and thus improve diffusion. The effect of water content on the solubility of carbon dioxide in a DES has been investigated only in a few studies. Computational methods for the investigation of the phase dynamics and mechanisms occurring in the systems are highly needed, as well as research on their behavior and properties in real applications. DESs are known to be solvents with satisfying CO_2_ capacity and the potential for implementation in industrial processes in the near future. It is highly desirable to focus on DESs for greener applications and to conduct further analyses on the technical and economic aspects of DES utilization. Although several DESs have been broadly studied, many more studies are still needed, especially regarding functional DESs. Future research should focus on (1) designing new, cheap, and environmentally friendly DESs with high CO_2_ capacity and low viscosity to make them attractive in gas separation; (2) technoeconomic research on the use of DESs in CO_2_ separation; (3) the regeneration of DESs used for CO_2_ capture; (4) the prediction of their properties and CO_2_ absorption capacity; and (5) the impact of water content on the DES properties, CO_2_ absorption capacity, and separation parameters.

## Figures and Tables

**Table 1:** Types of deep eutectic solvents.

Type	General Formulas	Examples
I	Cat^+^X^−^:metal halides	1:2 ChCl: FeCl_3_
II	Cat^+^X^−^:metal halides hydrate	1:2 ChCl:CrCl_3_·6H_2_O
III	Cat^+^X^−^:HBD	1:2 ChCl:H_2_NCONH_2_
IV	Metal chloride:HBD	1:3 ZnCl_2_:CH_3_CONH_2_
V	Nonsalt HBA:HBD	1:1 Citric acid:sucrose
VI	API as HBA or HBD (THEDES)	1:1 ChCl:phenylacetic acid
VII	Amino acid as HBA or HBD (AADES)	1:1 Betaine:L-histidine

## Data Availability

Data sharing is not applicable to this article.
